# Monitoring Adult Subglottic Stenosis With Spirometry and Dyspnea Index: A Novel Approach

**DOI:** 10.1177/01945998211060817

**Published:** 2021-11-23

**Authors:** Eleftherios Ntouniadakis, Josefin Sundh, Mathias von Beckerath

**Affiliations:** 1 Department of Ear Nose and Throat, Faculty of Medicine and Health, Örebro University, Örebro, Sweden; 2 Department of Respiratory Medicine, Faculty of Medicine and Health, Örebro University, Örebro, Sweden; 3 Department of Clinical Sciences, Intervention and Technology, Karolinska Institutet, Karolinska University Hospital, Stockholm, Sweden

**Keywords:** subglottic stenosis, peak expiratory flow, expiratory disproportion index, dyspnea index, functional assessment

## Abstract

**Objective:**

The aim was to examine the correlations among the anatomic Cotton-Myer classification, pulmonary function tests (PFTs), and patient-perceived dyspnea or dysphonia in patients with subglottic stenosis and identify measurements accurately reflecting treatment effects.

**Study Design:**

Prospective cohort study.

**Setting:**

Tertiary referral center.

**Method:**

Fifty-two adults receiving endoscopic treatment for isolated subglottic stenosis were consecutively included. Correlations were calculated among the preoperative Cotton-Myer scale, PFTs, the Dyspnea Index (DI), and the Voice Handicap Index. Receiver operating characteristic curves were determined for PFT, DI, and Voice Handicap Index pre- and postoperative measurements.

**Results:**

The Cotton-Myer classification correlated weakly with peak expiratory flow (*r* = −0.35, *P* = .012), expiratory disproportion index (*r* = 0.32, *P* = .022), peak inspiratory flow (*r* = −0.32, *P* = .022), and total peak flow (*r* = −0.36, *P* = .01). The DI showed an excellent area under the curve (0.99, *P* < .001), and among PFTs, the expiratory disproportion index demonstrated the best area under the curve (0.89, *P* < .001), followed by total peak flow (0.88, *P* < .001), peak expiratory flow (0.87, *P* < .001), and peak inspiratory flow (0.84, *P* < .001). Patients treated endoscopically with balloon dilatation showed a 53% decrease in expiratory disproportion index (95% CI, 41%-66%; *P* < .001) and a 37% improvement in peak expiratory flow (95% CI, 31%-43%; *P* < .001).

**Conclusion:**

Expiratory disproportion index or peak expiratory flow combined with DI was a feasible measurement for the monitoring of adult subglottic stenosis. The percentage deterioration of peak expiratory flow and increase in expiratory disproportion index correlated significantly with a proportional percentage increase in DI.

Subglottic laryngotracheal stenosis in adults is an uncommon mucosal scarring affecting the upper airway. With an incidence of <1 per million,^1,2^ this condition might be the result of systemic inflammatory and autoimmune disorders (ie, granulomatosis with polyangiitis, rheumatoid arthritis) or triggers of mucosal injury, whether apparent (eg, endotracheal tube) or obscure (eg, laryngopharyngeal reflux). However, in some patients the etiology remains unclear (idiopathic).^3,4^ The nonspecific constellation of symptoms, including gradual deterioration of exertional breathing, wheezing, stridor, dysphonia, or coughing, is frequently misinterpreted as other respiratory diseases, often resulting in a diagnostic delay.^4-6^ The anatomic classification commonly used was first described by Myer et al^7^ and is based on the cross-sectional area of the stenotic part of the airway: grade I, up to 50% obstruction; grade II, from 51% to 70%; grade III, >70% to any detectable lumen; and grade IV, when there is a completely obliterated airway. Endoscopic procedures, such as dilatation or serial steroid injections under local anesthesia, are considered to be less invasive treatment modalities than open surgery, which maintain a patent airway without the need for tracheostomy but have a higher risk of relapse and recurring surgical treatment.^4,8-11^

Spirometry could simplify decision making about the timing of intervention during follow-up of adults with subglottic stenosis. The association of several spirometry values with laryngotracheal stenosis have been studied in recent decades, yet no consensus has been reached about the exact role of pulmonary function tests (PFTs) in preoperative evaluation and postoperative outcome monitoring.^12-19^ Empey^12^ was the first to propose that the expiratory disproportion index (EDI)—the ratio of force expiratory volume in 1 second (FEV_1_) to peak expiratory flow (PEF)—could be a useful instrument in the assessment of upper airway obstruction, and the author’s findings were confirmed thereafter by other studies.^13,15,16^ Others have advocated for the more convenient sole PEF,^15,16,18,19^ peak inspiratory flow (PIF),^15,19^ total peak flow (TPF, as the sum of PEF and PIF),^13,16^ or the more complex-to-calculate ratio of the area under the inspiratory and expiratory curve to forced vital capacity.^14^ Lately, PEF%—as the patient’s measured PEF divided by the predicted PEF for someone of the same age, height, and sex—was proposed as a standardized spirometry measurement to describe disease severity in patients with subglottic stenosis.^18,20^ Furthermore, Naunheim et al sought to identify associations between PEF% and specific questions from a range of quality of life instruments.^20^

According to the American Thoracic Society, breathlessness should be measured regardless of its cause, to be assessed properly. Ideally, the patient-reported outcome instrument used to evaluate symptoms should be carefully selected with respect to describing all 3 dyspnea domains: sensory experience, affective distress, and symptom burden.^21^ Despite the multiple dyspnea questionnaires available, the Dyspnea Index (DI) was the only one specifically developed for the evaluation of upper airway dyspnea.^22-24^ Breathing is a function closely related to the voice, sharing the same apparatus. The Voice Handicap Index (VHI) is a widespread instrument broadly used to assess dysphonia.^25^

The aims of this study were to investigate the correlations of the anatomic Cotton-Myer classification with spirometry measures, DI, and VHI; to assess the effects of interventions on these values; and to identify the relevant spirometry measures that could clinically be used to evaluate treatment and predict the need for intervention.

## Materials and Methods

### Study Group

Adult patients who presented with isolated subglottic stenosis requiring surgical intervention at the Ear Nose and Throat Department at Örebro University Hospital (a tertiary referral hospital in Sweden) between September 2016 and December 2020, were consecutively included in this study. Those patients with stenosis involving other areas of the airway such as the intrathoracic trachea, glottis or supraglottis, and narrowing of the airway caused by external pressure or tumors, were excluded.

The DI is a 10-item, 5-point Likert scale questionnaire ranging from 0 to 40 that measures upper airway dyspnea. The VHI is a 30-item, 5-point Likert scale instrument that measures aspects of voice disorders; it ranges from 0 to 120. For each measure, a higher cumulative score represents more severe dyspnea and dysphonia, respectively. The Swedish versions of the DI^24^ and VHI^25^ were administered to the study subjects visiting the outpatient clinic, and preoperative spirometry was planned and carried out in the Department of Clinical Physiology, either in this hospital or at the referring county hospitals. Expiratory and inspiratory maneuvers were conducted.

All study subjects were treated endoscopically within 1 month of the lung function test, undergoing dilatation of the stenotic part of the airway (up to 15 mm in women and 18 mm in men) using continuous radial expansion balloons (Boston Scientific Corporation), following radial incisions with cold steel or CO_2_ laser. Cotton-Myer grading in all patients was assessed intraoperatively through visual estimation of the airway obstruction by the first author. Spirometry was similarly obtained within 4 to 6 weeks postoperatively, while the VHI and DI were registered at the same time frame by mailing them to the patients.

The demographic data obtained from the study subjects included sex, age, body mass index, etiology, smoking history, the presence of diagnosed or self-reported gastroesophageal reflux disease, and history of tracheostomy at any age or intubation within 2 years prior to the date of diagnosis. The spirometry values registered pre- and postoperatively were FEV_1_, PEF, PIF, and forced inspiratory volume in 1 second (FIV_1_) before bronchodilation, and based on these values, EDI and TPF were calculated.

### Statistical Analysis

A Shapiro-Wilk test was performed to investigate the normality of the baseline characteristics, all variables, and outcomes. Normally distributed continuous variables were presented in mean and standard deviation, nonnormally distributed variables with median and interquartile range, and categorical variables as number and percentage.

The associations among the preoperative Cotton-Myer scale, spirometry values, DI, and VHI were calculated with Spearman’s correlation coefficient. These correlations are reported as very strong (0.90 < *r* < 1.00), strong (0.70 < *r* < 0.89), moderate (0.40 < *r* < 0.69), weak (0.10 < *r* < 0.39), or negligible (0.00 < *r* < 0.09).^26^ The effects of endoscopic treatment on spirometry values, DI, and VHI were investigated by performing a Wilcoxon signed rank test for nonnormally distributed variables and a paired *t* test for normally distributed variables.

Receiver operating characteristic (ROC) analyses for each spirometry measure, DI, and VHI were conducted between pre- and postoperative values to investigate the most appropriate indicator for predicting the need for endoscopic intervention. The preoperative measurements represented disease severity requiring endoscopic treatment, and the postoperative values were references for a normal airway. A ROC curve illustrates the trade-off between clinical sensitivity and specificity for every cutoff value of a diagnostic test. The area under the curve (AUC) represents the test’s discriminatory power, which is categorized as follows: excellent (0.90 < AUC < 1.00), good (0.8 < AUC < 0.89), fair (0.70 < AUC < 0.79), poor (0.60 < AUC < 0.69), and failure (0.50 < AUC < 0.59). The optimal cutoff value providing balanced sensitivity and specificity is defined as the point on the apex of the ROC curve, being the highest point of the vertical axis and farthest to the left on the horizontal axis.^27^

Since spirometry measurements are affected by several factors restricting the use of cutoff values in predicting the need for endoscopic intervention, the implementation of a normalized variable is inevitable. Regarding the postoperative measurement as the patient’s best achieved value, we calculated the percentage deterioration preoperatively for the variables showing the best AUC: (Δtest variable / test variable) × 100%, where *Δtest variable* indicates post- and preoperative difference and *test variable* is the postoperative outcome. As a result, the patient became his or her own control, eliminating any underlying comorbidities or other individual factors affecting spirometry, and a personalized functional evaluation of the airway was achieved.

The 1-sample *t* test was then performed to investigate the magnitude of this normalized percentage deviation from the patients’ best spirometry values. Moreover, a correlation between the percentage functional deterioration in PFT and the subjective experience of the airway compromise as expressed by ΔDI% was examined with a Spearman correlation coefficient, following normality testing.

SPSS Statistics version 25 (IBM Corp) was used for statistical analysis. Due to the large number of assessed variables, the Bonferroni equation of α/n = 0.05 was used to calculate the *P* value. As the number of assessed variables was 8, a *P* value of .006 was considered statistically significant.

This human study was performed in accordance with the Declaration of Helsinki and was approved by the Ethics Review Board in Uppsala (2016/193). All adult participants provided written informed consent to participate.

## Results

### Study Sample

The study group consisted of 52 subjects; the detailed demographic data are presented in **Table 1**. None of them reported comorbidities from the lower airway or the lungs; however, 3 had been prescribed steroid inhalers by general practitioners suspecting asthma prior to diagnosing subglottic stenosis. None of them had a tracheostomy at any age. Six patients had marginally positive autoimmune serology tests other than ANCA (antineutrophil cytoplasmic antibody) and inconclusive biopsies obtained from the stricture. They were all evaluated by rheumatologists, yet the findings were insufficient to advocate for an autoimmune disease. In 6 patients, the inspiratory maneuver was not conducted postoperatively.

**Table 1.** Demographic Data of the Study Population.^a^

	No. (%) or mean ± SD
Sex	
Male	4 (7.7)
Female	48 (92.3)
Age, y	56.5 ± 13.8
Body mass index	29.0 ± 6.7
Underweight: <18.5	1 (1.9)
Healthy weight: 18.6-24.9	16 (30.8)
Overweight: 25-29.9	16 (30.8)
Obese: 30-39.9	16 (30.8)
Morbid obesity: >40	3 (5.8)
Smoking	
Current smoker	3 (5.8)
Never smoker	45 (86.5)
Former smoker	4 (7.7)
Etiology	
Idiopathic	41 (78.8)
Granulomatosis with polyangiitis	1 (1.9)
Positive autoimmune serology–ANCA negative	6 (11.5)
Rheumatoid arthritis	3 (5.8)
Prolonged intubation	1 (1.9)
Intubation history within 2 y prior to diagnosis setting	
Positive	15 (28.8)
Negative	37 (71.2)
Gastroesophageal reflux disease	
Positive	16 (30.8)
Negative	36 (69.2)
Diabetes	
Positive	6 (11.5)
Negative	46 (88.5)
Cotton-Myer preoperatively	
I	7 (13.5)
II	30 (57.7)
III	15 (28.8)

Abbreviation: ANCA, antineutrophil cytoplasmic antibody.

a Normally distributed continuous variables are presented as mean ± SD and categorical variables as No. (%).

### Correlations Between Variables Preoperatively

The Cotton-Myer classification showed a marginally significant weak correlation with PEF, EDI, PIF, and TPF, whereas the DI and VHI did not correlate with the spirometry measures or with the Cotton-Myer scale (**Table 2**).

**Table 2.** Pearson Correlation Coefficients: Preoperative Cotton-Myer Classification, Spirometry, DI, and VHI.

	FEV_1_	PEF	PIF	FIV_1_	TPF	EDI	Cotton-Myer	DI	VHI
Cotton-Myer	–0.21	–0.35^a^	–0.32^b^	–0.24	–0.36^c^	0.32^b^	—	0.24	–0.01
DI	0.10	–0.14	–0.94	0.02	–0.16	0.26	0.24	—	0.19
VHI	–0.21	–0.24	–0.25	–0.35	–0.24	0.11	0.01	0.19	—

Abbreviations: DI, Dyspnea Index; EDI, expiratory disproportion index; FEV_1_, forced expiratory volume in 1 second; FIV_1_, forced inspiratory volume in 1 second FIV_1_; PEF, peak expiratory flow; PIF, peak inspiratory flow; TPF, total peak flow; VHI, Voice Handicap Index.

a *P* = .012.

b *P* = .022.

c *P* = .010.

### Impact of Endoscopic Dilatation

The Shapiro-Wilk test of normality confirmed the null hypothesis of normally distributed data for PEF, PIF, FIV_1_, and EDI. There was a significant change between the pre- and postoperative values in all investigated variables (**Table 3**).

**Table 3.** Endoscopic Balloon Dilatation Treatment Effects in Different Spirometry Measurements, DI, and VHI.^a^

	Preoperative	Postoperative
FEV_1_, L	2.3 ± 0.7	2.6 ± 0.6
PEF, L/s	3.9 ± 1.4	6.2 ± 1.5
PIF, L/s	2.7 ± 0.9	4.0 ± 1.0
FIV_1_, L	2.2 ± 0.7	2.9 ± 0.7
TPF	6.6 ± 2.2	10.1 ± 2.3
EDI	0.60 (0.30)	0.42 (0.08)
DI	31.5 (10)	4 (9)
VHI	24.5 (41)	7.5 (19)

Abbreviations: DI, Dyspnea Index; EDI, expiratory disproportion index; FEV_1_, forced expiratory volume in 1 second; FIV_1_, forced inspiratory volume in 1 second; PEF, peak expiratory flow; PIF, peak inspiratory flow; TPF, total peak flow; VHI, Voice Handicap Index.

a Values are presented as mean ± SD or median (interquartile range). Each pre- and postoperative comparison: *P* < .001.

### Spirometry, DI, and VHI Predicting Endoscopic Intervention

**Figure 1.**
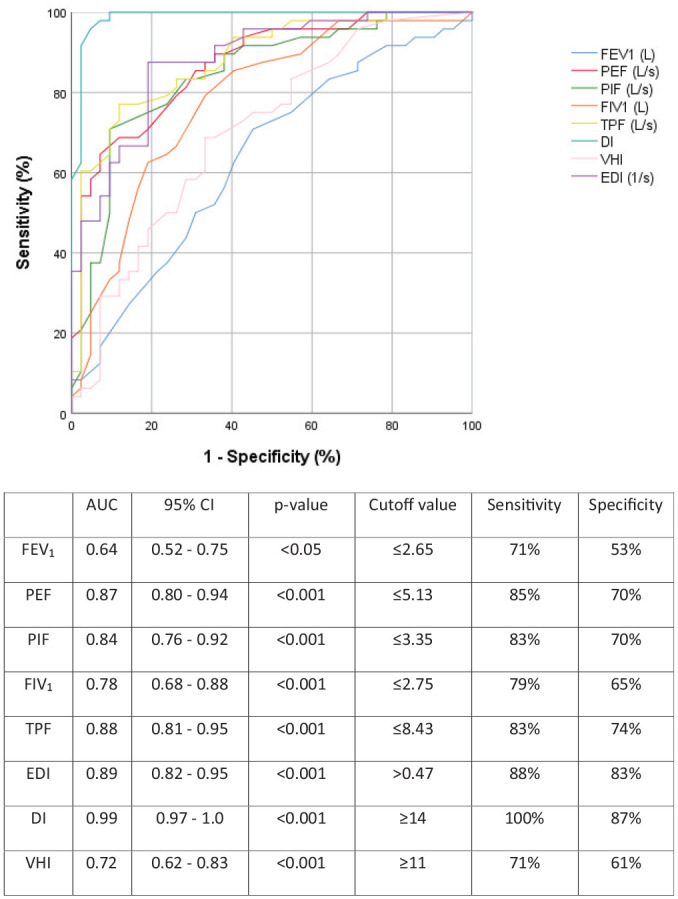
Receiver operating characteristic analysis, areas under the curve (AUCs), and cutoff values for the spirometry measures, DI, and VHI. DI, Dyspnea Index; EDI, expiratory disproportion index; FEV_1_, force expiratory volume in 1 second; FIV_1_, forced inspiratory volume in 1 second; PEF, peak expiratory flow; PIF, peak inspiratory flow; TPF, total peak flow; VHI, Voice Handicap Index.

**Figure 2.**
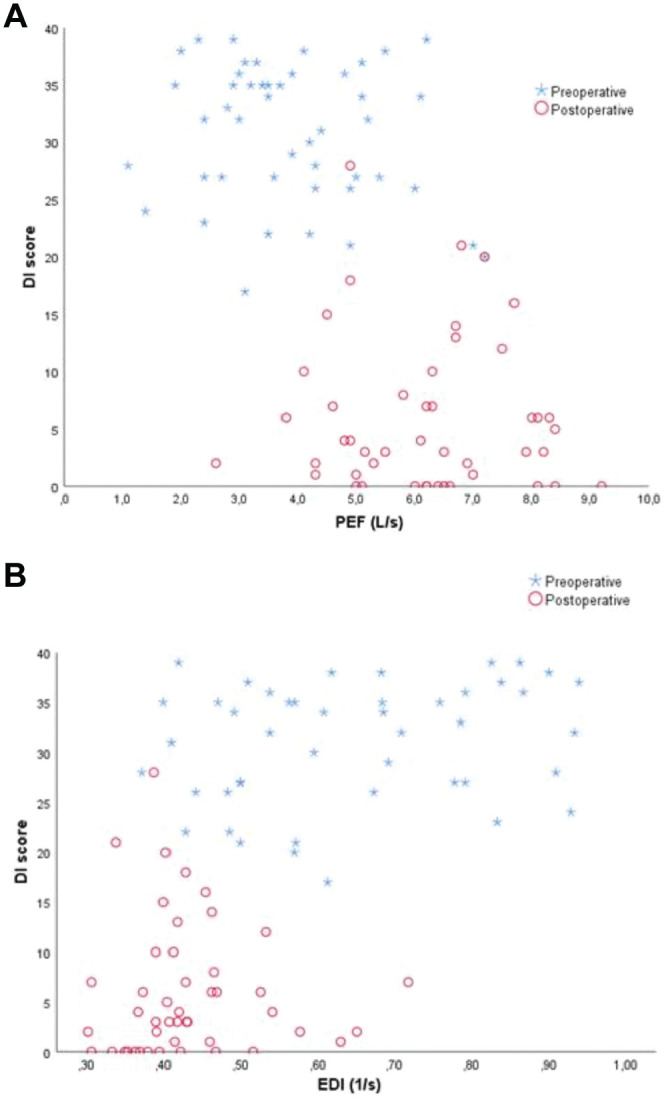
Scatterplot pairing pre- and postoperative observations: (a) DI-PEF and (b) DI-EDI. DI, Dyspnea Index; EDI, expiratory disproportion index; PEF, peak expiratory flow.

We found an excellent AUC for the DI, with an optimal cutoff value ≥14, showing 100% sensitivity and 87% specificity, while only a fair AUC for the VHI. Regarding the PFTs investigated, the EDI demonstrated the best area under the ROC curve, followed in order by TPF, PEF, and PIF. **Figure 1** presents a detailed analysis of the ROC curve characteristics, including optimal cutoff points maximizing sensitivity and specificity for all the studied variables. **Figure 2** illustrates the interaction between pre- and postoperative values of the DI combined with PEF and EDI, respectively, in each study subject.

Unlike the percentage PEF decrease (ΔPEF%), which was normally distributed, the percentage EDI (ΔEDI%) and DI (ΔDI%) changes were not. As such, Spearman’s *r* was chosen to investigate the correlations among these variables, indicating significant moderate positive associations between ΔDI% and ΔPEF% (*r* = 0.46, *P* < .001) and ΔDI% and ΔEDI% (*r* = 0.49, *P* < .001). In **Table 4**, we present the percentage deterioration of PFTs from the patients’ best measurements indicating the need of endoscopic treatment.

**Table 4.** Preoperative Percentage Deterioration in Spirometry Measures Requiring Endoscopic Treatment vs the Patient’s Best Postoperative Spirometry Score.^a^

	Δ Value, %^b^		
	Mean	SD	95% CI
EDI	53	42	41-66
TPF	34	18	29-40
PEF	37	20	31-43
PIF	30	19	24-36

Abbreviations: EDI, expiratory disproportion index; PEF, peak expiratory flow; PIF, peak inspiratory flow; TPF, total peak flow.

a Each row: *P* < .001.

b Calculated as follows: (post- and preoperative difference/postoperative score) × 100%.

## Discussion

The primary findings of this study suggest that an objective functional assessment based on EDI, TPF, PEF, or PIF, combined with a subjective one based on the DI, is a promising noninvasive way to monitor the treatment effect and to follow up airway deterioration in patients with subglottic stenosis. The PFT measurements and DI alone do not seem to represent the severity grade of stenosis, since patients estimate dyspnea individually and pulmonary function is difficult to compare even with normalized values among individuals of the same age, height, and weight. The data from this study suggest that there is a significant moderate correlation between ΔDI% and spirometry measurements such as ΔPEF% and ΔEDI%. Consequently, the DI and PFT assess different aspects of airway compromise and seem to be related and complete each other; so, in this study, a minimum of 30% deterioration in PEF or a 40% increase in EDI indicated the need for endoscopic treatment.

The Cotton-Myer classification was established in pediatric populations where the extent of airway obstruction was assessed by comparing the outer diameter of an endotracheal tube tolerating normal leak pressure while passing the stenotic part of the airway with an expected age-appropriate tube.^7,28^ In accordance with other studies,^18,19,29^ the detection of weak correlations among Cotton-Myer grading, pulmonary function deterioration, and patient-reported breathlessness reflected a discrepancy between the anatomic and perceived or functional status commonly observed in everyday clinical practice, where patients with substantial obstruction experience relatively mild distress and vice versa.^17,19^ Computational fluid dynamic studies claim that anatomic variations such as the axial position of stenosis or the cross-sectional subglottic area may affect the perception of breathlessness.^30,31^

The impact of subglottic stenosis in PFTs has been studied over the past few decades, although they are not routinely used in assessment and follow-up. Most of the test measurements improve postoperatively^14,15,19^; yet, the EDI was found to be the most reliable parameter in diagnosing subglottic stenosis^12,13^ and in monitoring treatment outcomes,^15-19^ something supported by our findings. PEF^16,18,20^ and PEF%^18,20^ are also regarded as decent and convenient measurements of airway deterioration in follow-up subglottic stenosis. However, neither cutoff PEF values nor PEF% can incorporate the dissimilarity in spirometry values among the patients, related to general physical status or individual comorbidities such as underlying cardiopulmonary issues. Crosby et al^17^ were the first to show that spirometry changes should be assessed in comparison with the patient’s highest values, integrating patient-specific factors affecting PFT, such as physical condition, breathing requisites, medications, or cardiopulmonary comorbidities.

The approach of this study to using ROC curves was not intended to provide cutoff values to detect a relapse of the condition but to indicate the measurement of confidence when assessing subglottic stenosis. Acknowledging that the cutoff points set in this study were quite low compared to the normal values for age and sex, we note that it could be advantageous to opt for higher sensitivity at the cost of lower specificity, considering that subglottic stenosis is a rare condition.^27^ It is the factors affecting the subjective experience of breathlessness that differ among individuals that are impossible to assess by spirometry alone, such as physical condition, the extent and need of daily physical activity, other comorbidities, or behavioral factors related to dyspnea.^21^

The DI has been found to be a useful instrument for assessing upper airway dyspnea, and it should be used complementarily to measure only the subjective aspect of breathlessness.^22,24^ The results from this study advocate that a DI score exceeding 14 could be the ideal supplement to PEF and EDI changes, acting synergistically, in the assessment of a suspected relapse of subglottic stenosis. **Figure 2** illustrates the effect of combining the DI with PFTs in the assessment of subglottic stenosis, as DI refines the diagnostic value of solitary spirometry measurements by formulating distinct clusters between pre- and postoperative spirometry observations. Conversely, the VHI does not seem to have a role in the diagnosis and follow-up of subglottic stenosis. Future longitudinal studies with larger sample sizes could be performed to investigate whether this approach is proper.

The ΔPEF%, although slightly inferior to the ΔEDI%, appears to be an easy value to obtain, without the need to extract it from other measurements (eg, EDI) or to perform uncommon inspiratory maneuvers. A peak flow meter is an inexpensive handheld device recommended to follow up treatment effects in conditions of the lower airway.^32^ This study suggests a similar strategy for patients treated endoscopically for subglottic stenosis, where self-monitoring of the proportional PEF percentage reduction as compared with the postoperative value when the airway is normal (ΔPEF%) could be a cost-effective and convenient way to indicate early airway deterioration, providing a standardized value that allows for comparisons among patients in clinical practice and for research purposes. Upon establishing objective signs of worsening, the patient should be encouraged to reestablish contact with a health care provider. If an early diagnosis of a relapse could be confirmed, it would allow for noninvasive treatment modalities, such as a series of intralesional steroid injections.^11^

### Strengths and Limitations

The main strengths of this study were the unique population of a very uncommon condition and the investigation of all available lung function tests, combined with subjective assessments in these patients. The study results demonstrated the association of the individual experience of dyspnea in subglottic stenosis with the functional deterioration and how they could be appropriately measured. This is equally important to history taking and the physical examination according to the recommendations about the assessment of dyspnea from the American Thoracic Society.^21^

The main limitation of this study was the small sample size, which could be addressed in the future by conducting multicenter studies. To minimize random error, all of our patients underwent PFTs in the Department of Clinical Physiology, in our hospital and in the referring hospitals, with the purpose of obtaining a standardized procedure in accordance with the guidelines from the American Thoracic Society/European Respiratory Society.^33^ However, the inspiratory maneuver is not conducted routinely by the operators, leading to systematic error possibly favoring the expiratory part of the test and random error inevitably related to the procedure itself. The lack of systematically recorded longitudinal spirometry tests to investigate a relapse pattern may lead to selection bias for patients having significant deterioration in breathing as compared with those with only moderate dyspnea and slight obstruction and a relapse in early stages.

## Conclusion

Spirometry, particularly ΔPEF% and ΔEDI%, combined with DI could be a reliable way to functionally assess upper airway dyspnea due to subglottic stenosis. We recommend an individualized and patient-centered follow-up by monitoring the ΔPEF% with a PEF meter.
